# Characterization and insight mechanism of an acid-adapted β-Glucosidase from *Lactobacillus paracasei* and its application in bioconversion of glycosides

**DOI:** 10.3389/fbioe.2024.1334695

**Published:** 2024-01-24

**Authors:** Yufeng Xie, Xinrui Yan, Changzhuo Li, Shumei Wang, Longgang Jia

**Affiliations:** ^1^ College of Food Science and Engineering, Harbin University, Harbin, China; ^2^ College of Food Science and Engineering, Tianjin University of Science and Technology, Tianjin, China

**Keywords:** β-glucosidase, acid-adapted, enzymatic property, molecular dynamics simulations, bioconversion

## Abstract

**Introduction:** β-glucosidase is one class of pivotal glycosylhydrolase enzyme that can cleavage glucosidic bonds and transfer glycosyl group between the oxygen nucleophiles. *Lactobacillus* is the most abundant bacteria in the human gut. Identification and characterization of new β-glucosidases from *Lactobacillus* are meaningful for food or drug industry.

**Method:** Herein, an acid-adapted β-glucosidase (LpBgla) was cloned and characterized from *Lactobacillus paracasei*. And the insight acid-adapted mechanism of LpBgla was investigated using molecular dynamics simulations.

**Results and Discussion:** The recombinant LpBgla exhibited maximal activity at temperature of 30°C and pH 5.5, and the enzymatic activity was inhibited by Cu^2+^, Mn^2+^, Zn^2+^, Fe^2+^, Fe^3+^ and EDTA. The LpBgla showed a more stable structure, wider substrate-binding pocket and channel aisle, more hydrogen bonds and stronger molecular interaction with the substrate at pH 5.5 than pH 7.5. Five residues including Asp45, Leu60, Arg120, Lys153 and Arg164 might play a critical role in the acid-adapted mechanism of LpBgla. Moreover, LpBgla showed a broad substrate specificity and potential application in the bioconversion of glycosides, especially towards the arbutin. Our study greatly benefits for the development novel β-glucosidases from *Lactobacillus*, and for the biosynthesis of aglycones.

## Introduction

β-glucosidase (Bgla), or β-D-Glucoside glucohydrolase (3.2.1.21) is a dominant class of pivotal glycosylhydrolase enzyme that has been well-characterized in catalyzing the selective cleavage of glucosidic bonds and transferring glycosyl group between the oxygen nucleophiles (Bhatia et al., 2002). Bgla usually hydrolyzes the terminal, non-reducing β-D-glucosyl residues to release β-D-glucose from lots of glucoconjugates including glucosides, oligosaccharides and *1*-*O*-glucosyl esters (Godse et al., 2021). Therefore, the wide range of substrates in nature contributes the ubiquitous applications of Bgla in almost all aspects of life, for example, in lots of biotechnological and industrial applications containing degradation of structural and storge polysaccharides (Bhatia et al., 2002), hydrolysis of pharmaceutical ingredients (Xiao et al., 2014), production of sugar or fuel ethanol from lignocellulosic biomass (Singhania et al., 2017), or in many crucial biological processes including cellular signaling, host-pathogen interactions, et al. (Bhatia et al., 2002).

Bgla is commonly distributed in almost all living organisms from simple bacteria to highly evolved mammals, and perform a variety of physiological roles in target host depending on their location or functions (Collins et al., 2007; Fan et al., 2011). Filamentous fungi and bacteria are known as abundant Bgla resources, in which, Bgla is mainly acted as cellulase enzyme and responsible for the catalysis of short chain oligosaccharides and cellobiose to produce glucose (Molina et al., 2016). For example, Yan et al. identified a novel Bgla from *Paecilomyces thermophila*, and heterologous expressed and characterized in *Pichia pastoris*, high enzymatic activity and yield of Bgla were obtained by high cell-density fermentation (Yan et al., 2012). A cold-adapted Bgla from a psychrotolerant bacterium *Micrococcus antarcticus* was cloned, over-expressed and characterized using the *E. coli* expression system, the enzyme exhibited maximal activity at 25°C and pH 6.5 (Fan et al., 2011). We also developed an acid-stable Bgla from *Aspergillus aculeatus*, characterized the enzymatic properties and probed the involved acid-denaturation mechanism using molecular dynamics simulations (Li Y. et al., 2018).

Many complex carbohydrates or degradation-resistant plant cell wall glycans from our diets can not be digested by human enzymes (Martens et al., 2011; Ndeh et al., 2017). Symbiotic bacteria inhabiting in the human gut can utilize these polysaccharides to produce low molecular weight carbohydrates or absorbable short chain fatty acids (SCFAs) (Martens et al., 2011). For example, *Bacteroidetes thetaiotaomicron* in human gut can utilize and depolymerize the large mannan oligosaccharides to generate mannose by the periplasmic enzymes (Cuskin et al., 2015). Moreover, the elaborate and highly specific enzyme system in gut bacteria can depolymerize the highly complex glycans, polysaccharide rhamnogalacturonan-II, by cleaving all but 1 of its 21 distinct glycosidic linkages (Ndeh et al., 2017). *Liquorilactobacillus satsumensis* isolated from water kefir could use the carbohydrates to produce low molecular weight glucans, and the hydrolyzed products showed prebiotic properties that could promote the abundance of *Bacteroidetes* and increase the contents of SCFAs, including acetate, proionate and butyrate (Tan et al., 2022). *Lactobacillus* are one of the most abundant bacteria in the human gut, so, *Lactobacillus* may have rich and robust glycosidase enzyme system. Identification and characterization of new glycosidases from *Lactobacillus* are meaningful for food or drug industry. Although the Bgla is one of the most widely used glycosidases, there are few Bgla have been identified or deeply studies from the *Lactobacillus*. Therefore, in this study, a novel *Bgla* gene from *Lactobacillus paracasei* TK1501 (*LpBgla*) was cloned and high-efficiently expressed in *E. coli*. After purified using Ni-NTA column, the enzymatic properties of LpBgla enzyme including optimum temperature, pH, stability, enzyme kinetics, and substrate specificity are systemically investigated. Furthermore, the insight acid-adapted mechanism, and the key residues involved in the catalysis of Bgla were investigated using molecular dynamics simulations. We think this work has great significance for the theoretical research and practical application.

## Materials and methods

### Materials

DNA Purification Kit, Quick Plasmid MiniPrep Kit were purchased from Thermofisher Scientific (Waltham, MA, United States). DNA polymerase, RNase, DNA Ligation Kit Ver.2, restriction enzymes of *Hin*dIII and *Xho*I were purchased from Takara (Dalian, China). Ni-NTA column and resin were purchased from Qiagen (Hilden, Germany). The substrates of β-*p*-Nitrophenyl-β-D-glucuronide (β-*p*NPG), geniposide, salicin, polydatin, heptaside and arbutin were purchased from Sigma-Aldrich (St. Louis, MO, United States). Other reagents or chemicals with high purity were derived from local sources unless noted.

### Strains and plasmids

*Escherichia coli* (*E. coli*) JM109 and BL21(DE3) were cultivated using Luria-Bertani (LB) media and stored in our lab. *E. coli* JM109 was used as the cloning host, and BL21(DE3) was used as expression host of *LpBgla* gene. *Lactobacillus paracasei* TK1501 was screened from Chinese traditional fermented food, cultured using MRS media and stored in our lab. Plasmid used in cloning and expression of *LpBgla* gene was pET28a vector (Novagen, Darmstadt, Germany).

### Cloning, expression and purification of LpBgla

The cloning, expression and purification of recombinant LpBgla were following and modified from previous reported methods (Jia et al., 2018; Shang et al., 2022). The *LpBgla* gene was amplified using the *Lactobacillus paracasei* TK1501 genome DNA as template, and the specific primers were LpBgla-F (5′- GGA​ATT​CCA​TAT​GGG​GGT​GGT​AGT​ATC​TAA​CTT​TC-3′) and LpBgla-R (5′- CCC​AAG​CTT​TTA​TCT​AAA​AAT​CAT​TTT​CAC​ATA​CTG​ATG-3′). Then the fragment of *LpBgla* was double digested using *Hin*dIII and *Xho*I enzymes, and ligated into the pET28a vector with same restriction enzyme sites to construct the expression plasmid. After identified by DNA sequencing, the expression plasmid was named as pET28a-*LpBgla*.

The pET28a-*LpBgla* plasmid was transferred into the expression host *E. coli* BL21(DE3) to prepare recombinant strain. The colonies were identified by PCR method using LpBgla-F/LpBgla-R primers, and the positive recombinant strain was named BL21-*LpBgla*. Single colony of BL21-*LpBgla* was selected and cultured in 5 mL LB medium at 37°C overnight. Then, 2% (v/v) of the overnight-incubated bacteria solution was inoculated into 100 mL fresh LB medium, and incubated at 37°C until the OD_600_ was to 0.6–0.8. 0.5 mM isopropyl-β-D-thiogalactopyranoside (IPTG) was added into the solution to induce the overexpression of LpBgla at 16°C. After cultured for 16–20 h, the BL21-*LpBgla* cells were collected by centrifuging at 12,000 rpm 10 min, and then resuspended in lysis buffer (20 mM Tris, pH 7.4, 20 mM imidazole, 0.5 M NaCl, 1 mM DTT). A sonication process was employed to break BL21-*LpBgla* cells for the releasing of intracellular proteins. Subsequently, the bacteria solution was centrifuged at 12,000 rpm at 4°C for 30 min, and the supernatant was collected. We used the Ni-NTA method to purify the His-tagged LpBgla enzyme. Firstly, the supernatant of BL21-*LpBgla* sonication solution was gently mixed with the agarose resin and combined for 1 h at room temperature. Then the mixture solution was trapped on a column, and flowed out naturally through gravity. The resin was washed by wash buffer (20 mM Tris, pH 7.4, 40 mM imidazole, 0.5 M NaCl, 1 mM DTT), and the target LpBgla protein was eluted by elution buffer (20 mM Tris, pH 7.4, 400 mM imidazole, 0.5 M NaCl, 1 mM DTT). SDS-PAGE was used to identify the proteins during the purification process. At last, the purified LpBgla protein was dialyzed against Tris (50 mM, pH 7.4) and used for enzymatic properties characterization.

### β-glucosidase activity measurement

The β-glucosidase activity was assessed following previous method using β-*p*NPG as substrate (Li Y. et al., 2018). Briefly, 0.1 mL purified enzyme sample (0.7 mg/mL) was added into 0.5 mL working solution with 5 mM β-*p*NPG. After incubated at 30°C for 30 min, the reaction was terminated by addition 0.25 mL 0.2 M Na_2_CO_3_. Then the absorbance of the samples was measured using a plate reader at 410 nm. One unit (U) of LpBgla activity was designated as the amount of enzyme that produce 1 μmol p-nitrophenol per minute. The concentration of LpBgla was measured using Nanodrop 2000 (Thermo Fisher Scientific, Wilmington, United States).

### Characterization of the LpBgla

In order to investigate the optimal temperature of the LpBgla activity, we measured the enzyme activity using above standard reaction method at various temperatures of 20, 30, 40, 50, 60°C and 70°C. To access the thermostability of LpBgla, the enzyme was incubated at 20, 30, 40, 50°C and 60°C for 10, 30, 60, 90 and 120 min, the residual enzyme activity was tested using standard protocol. The initial LpBgla enzyme activity was defined as 100%.

The optimal pH of LpBgla activity was also probed by the above standard method, that is, the enzymatic reaction was performed at 30°C at different pH from 4.0 to 8.0. Meanwhile, pH stability was measured by treating the enzyme at different pH from 4.0 to 8.5 at 4°C for 60 min, and the residual enzyme activity was tested using standard protocol. The maximum LpBgla enzyme activity was defined as 100%.

Moreover, the impact of metal ions and EDTA on the activity of LpBgla was investigated. 5 mM metal ions including Mg^2+^, Mn^2+^, Fe^2+^, Ca^2+^, Zn^2+^, Cu^2+^, Fe^3+^ and EDTA were individually added into the reaction system. Then the LpBgla activity was measured using standard method at 30°C and pH 5.5. The relative enzyme activity of LpBgla in the absence of metal ions was defined as 100%.

### Enzymatic kinetic analysis

The kinetic parameters of LpBgla were analyzed by measuring the enzyme activity at the optimum reaction conditions using different concentrations of substrate from 0.5 to 15 mM. The *K*_*m*_ and *V*_max_ values were determined by fitting the data to the Michaelis-Menten equation, and the *k*_*cat*_ was calculated by *k*_*cat*_ = *V*_max_/(Cenc.). All the data were processed using GraphPad prism 8.0 software.

### Molecular docking and molecular dynamic (MD) simulations

The crystal structure of LpBgla was predicted and obtained from AlphaFold2 database (UniProt ID: Q03B82). 3D structure of β-pNPG was download from PubChem (http://pubchem.ncbi.nlm.nih. gov), and optimized by MOPAC (Stewart, 1990). AutoDock 4.2.6 and AutoDockTools 1.5.6 were used to conduct the molecular docking (Morris et al., 2009; Maier et al., 2015). The grid dimensions were set as 60 Å × 60 Å × 60 Å, and grid center was defined at x = 2.014, y = −13.655, z = 16.129.

To set the different pH values, the LpBgla was firstly treated using the online tool H++ (http://newbiophysics.cs.vt.edu/H++/) to calculate the pKa of each residue, then pH 5.5 or pH 7.5 environment was given to the enzyme-substrate complex, and a new pdb profile was obtained for the MD simulations. The MD simulations were performed using the GROMACS 2018.4 simulation package (Jorgensen et al., 1983), and with the force field of AMBER14SB (Mahoney and Jorgensen, 2000). Firstly, the LpBgla and β-*p*NPG substrate complexes were placed in a square box. Equal molecule of positive Na^+^ and negative Cl^−^ were added to substitute a part of water. Rational spacing among LpBgla-β-*p*NPG complex and water molecules were conducted using steepest descent minimization method. Initial random velocity was made by the Maxwell distribution, and the NVT simulation time was defined as 1 ns. All the covalent bonds relating to the hydrogen atoms were constrained by LINCS algorithm (Hess, 2008). The long-range electrostatic interactions were calculated using Particle Mesh Ewald (PME) method with a grid size of 10 Å (Darden et al., 1993). The temperature was set as 333 K by V-rescale (Berendsen et al., 1984), and pressure was maintained as 1 bar under isothermalisostatic (NPT) ensemble by Parrinello-Rahman (Wang et al., 2022) method. MD simulations were carried out for 100 ns. Based on the MD trajectory, root-mean-square deviation (RMSD), root-mean-square fluctuation (RMSF), radius of gyration (Rg) and solvent accessible surface area (SASA) values of LpBgla were calculated to analysis the structural stability with GROMACS package using *gmx rms*, *gmx rmsf*, *gmx gyrate* and *gmx sasa* programs, respectively. At last, Molecular Mechanics/Poisson Boltzmann Surface Area (MM/PBSA) was utilized to calculate the binding free energy between the LpBgla and β-*p*NPG (detailed method was deposited in Supporting Material). Herein, two MD simulation calculations were carried out: one run set the environment pH as the optimal pH of LpBgla, pH 5.5; and the other run set as an alkaline environment, pH 7.5.

### Substrate specificity and glycosides bioconversion of LpBgla

The catalytic specificity of LpBgla was measured with different substrates including geniposide, salicin, polydatin, esculin and arbutin. β-*p*NPG was used as the control substrate. Same to the β-glucosidase activity measurement, 0.1 mL purified LpBgla solution (0.7 mg/mL) was gently mixed with 0.5 mL 5 mM different substrates (dissolved in 0.1 mM PBS buffer, pH 5.5). The enzymic catalytic reaction was performed at 30°C for 30 min, and terminated by adding 0.25 mL 0.2 M Na_2_CO_3_ solution. The enzymatic activity was evaluated by detecting the production of glucose or aglycone using standard HPLC method (Yeom et al., 2012; Li Y. et al., 2018). Herein, one unit (U) enzyme activity was defined as the amount of LpBgla to produce 1 μmol glucose or aglycone per minute. Moreover, the kinetic parameters of LpBgla using each substrate were also analyzed using above methods.

β-Glucosidase can hydrolysis the β-glucosidic bond of glycosides to form aglycone and glucose. In order to analysis the bioconversion effects of LpBgla on glycosides, several different glycosides including geniposide, salicin, polydatin, esculin and arbutin were measured. The catalytic reaction was following the method of β-glucosidase activity measurement. Briefly, 0.1 mL purified LpBgla enzyme (0.7 mg/mL) was added into 0.5 mL working solution with 5 mM different glycoside samples. After incubated at 30°C for 12 h, the reaction was terminated by addition 0.25 mL 0.2 M Na_2_CO_3_. Then the reaction samples were detected by a High-performance liquid chromatography (HPLC) system using a reversed-phase chromatography NovaPak C18 column (250 × 4.6 mm, 5 μm, Waters, MA, United States) with a photodiode array detector (PDA-100, Dionex). The mobile phases were methanol with 0.1% acetic acid (phase A) and water with 0.1% acetic acid (phase B). The flow rate was 1 mL/min, and 10 μL of the samples were injected into the HPLC system. Other detailed HPLC parameters were following our previously reported method (Xie et al., 2022).

## Results and discussion

### Sequence identity analysis of LpBgla

**FIGURE 1:**
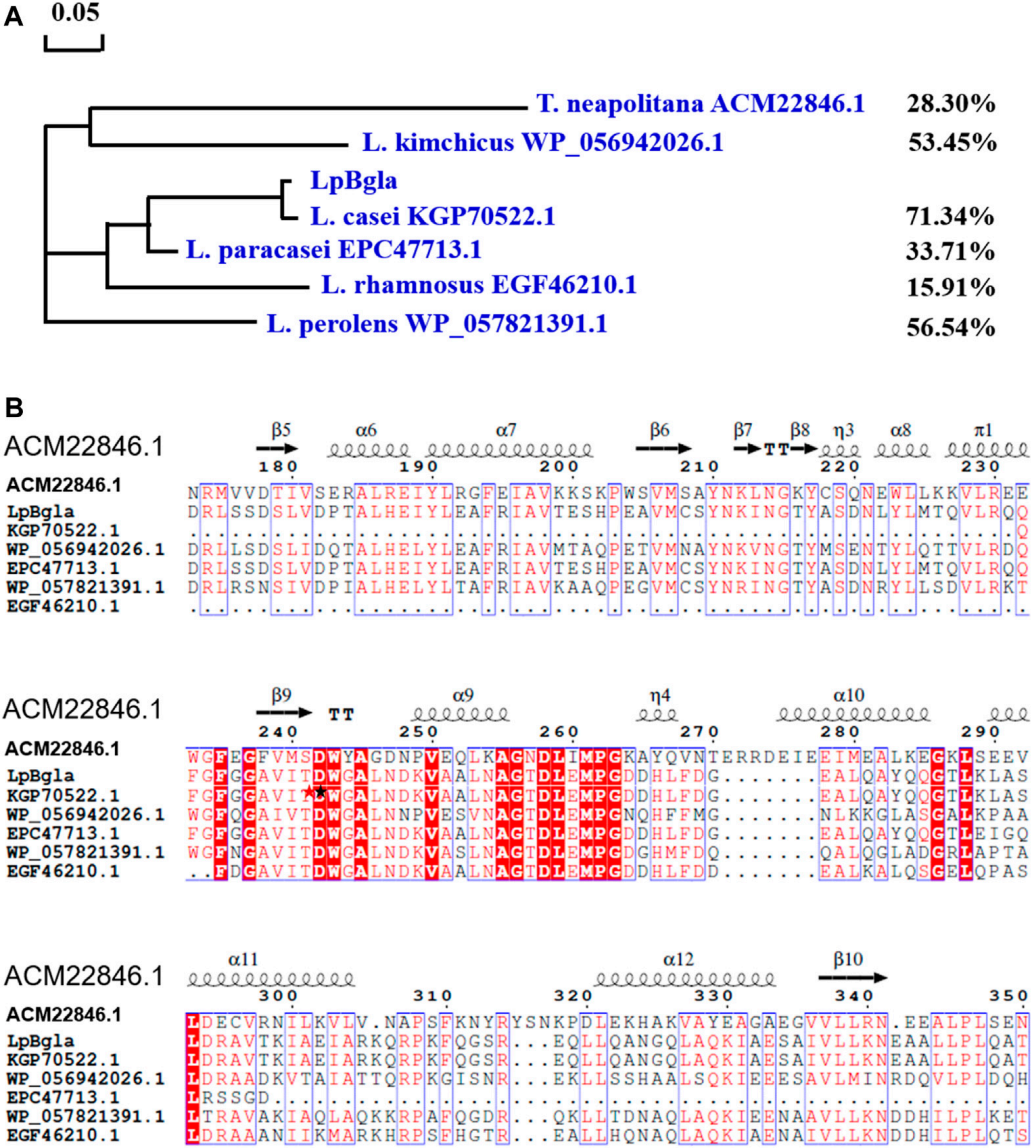
Sequence analysis of LpBgla with six Bglas from *Lactobacillus casei*, *Lactobacillus perolens*, *Lactobacillus kimchicus*, *Lactobacillus paracasei* and *Lactobacillus rhamnosus* and *Thermotoga neapolitana*. **(A)** Phylogenetic tree of LpBgla that constructed using MEGA 6.0. **(B)** Multiple alignment of LpBgla amino acid sequence with other six Bglas. The residues marked in red were strictly conserved, and boxed in blue were highly conserved. The secondary structure elements were labeled above the corresponding sequences.

In our previously reported study, the complete genome sequence of *Lactobacillus paracasei* TK1501 has been sequenced and deposited in the GenBank database (accession number: CP017716) (Xie et al., 2022). Based on the genome sequence of *Lactobacillus paracasei* TK1501, the GenBank locus of *LpBgla* was BKQ19_03070. Detailly, the gene of *LpBgla* is 2,388 bp, encodes 796 amino acids (Supplementary Table S1). Blast search in the GeneBank from NCBI showed that LpBgla exhibited the highest amino acid sequence identity of 71.34% with the Bgla from *Lactobacillus casei*. And the similarity of LpBgla with the Bglas from *Lactobacillus perolens*, *Lactobacillus kimchicus*, *Lactobacillus paracasei* and *Lactobacillus rhamnosus* were 56.54%, 53.45%, 33.71% and 15.91%. The structure and function of Bgla derived from *Thermotoga neapolitana* DSM 4359 (GenBank ID: ACM22846.1) has been analyzed in previously reported literature (Pozzo et al., 2010), which showed 28.30% amino acid sequence identity with LpBgla. The phylogenetic tree was constructed by using MEGA 6.0 biology software according to the blast sequence results, which showed that the LpBgla formed same branch, and was most closely related to β-glucosidase derived from *Lactobacillus casei* (Figure 1A).

Based on the blast results, the multiple sequence alignment of LpBgla was carried out using the online server Clustal X, and modified using the ESPript 3.0 (Supplementary Figure S1). Figure 1B showed the relative conserved part of the alignments, three strictly conserved fragments were identified including FxGxxxxDWxAxxxxV (residues 235–250), AGxDLxMPG (residues 255–263), and GxLxxxxL (residues 286–293). According to the crystal structure of ACM22846.1 from *T. neapolitana*, D242 and E458 were proved to be the essential catalytic residues, D58 and W243 were important for substrate accommodation or interaction (Pozzo et al., 2010), both of these two residues were conversed from the results of multiple sequence alignments. While in lots of other literature, two conserved residues of Glu located at the end of β-strand 4 and β-strand 7 were the general catalytic site for the β-glucosidases (Jeng et al., 2011; Kaenying et al., 2023). In line with the blast results, the identity of LpBgla with the other six Bglas ranged from 15% to 72%. Together with the results of the sequence comparison analysis, LpBgla was a novel Bgla family member.

### Cloning, expression and purification of the LpBgla

We designed the specific primers of *LpBgla* according to the complete genome sequence of *Lactobacillus paracasei* TK1501. The *LpBgla* gene was cloned using the genome DNA as template. As shown in Supplementary Figure S2A, a specific DNA band of 2,388 bp was observed in the gel, which in line with the theoretical molecular value of *LpBgla*. Then the *LpBgla* gene was cloned into the pET28a vector to construct the expression plasmid pET28a-*LpBgla*. The pET28a-*LpBgla* was identified using *Hin*dIII or *Xho*I single digestion, or *Hin*dIII and *Xho*I double digestion, respectively. Enzymic digestion results were consisted with the theoretical prediction, which indicated that the expression pET28a-*LpBgla* plasmid was successfully constructed (Supplementary Figure S2B). The pET28a-*LpBgla* plasmid was introduced into *E. coli* BL21 for heterologous expression of *LpBgla*. The positive transformants were identified using colony PCR, the results showed that the three picked transformants were true recombinants carrying the *LpBgla* gene (Supplementary Figure S2C). We renamed the recombinant *E. coli* BL21 that transformed with pET28a-*LpBgla* as BL21-*LpBgla*.

After induced by IPTG, the LpBgla enzyme was expressed in *E. coli* BL21. We respectively measured the β-glucosidase activity of intracellular and extracellular parts by standard methods. The activity of intracellular section was 48.3 U/mg, and extracellular section was 1.5 U/mg, suggesting that the recombinant LpBgla protein was mainly expressed in the intracellular part. Then the LpBgla was purified using Ni-NTA affinity chromatography, as shown in Supplementary Figure S2D, a ∼85 kDa band was observed in the gel, which was almost identical to the theoretical mess of LpBgla (about 85.6 kDa). The LpBgla was also further purified and identified using size-exclusion chromatography, which proved that this enzyme existed as monomeric state (Supplementary Figure S3). These results indicated that the LpBgla from *Lactobacillus paracasei* TK1501 was successfully cloned and heterologous expressed in *E. coli*.

### Enzymatic characterization of LpBgla

**FIGURE 2:**
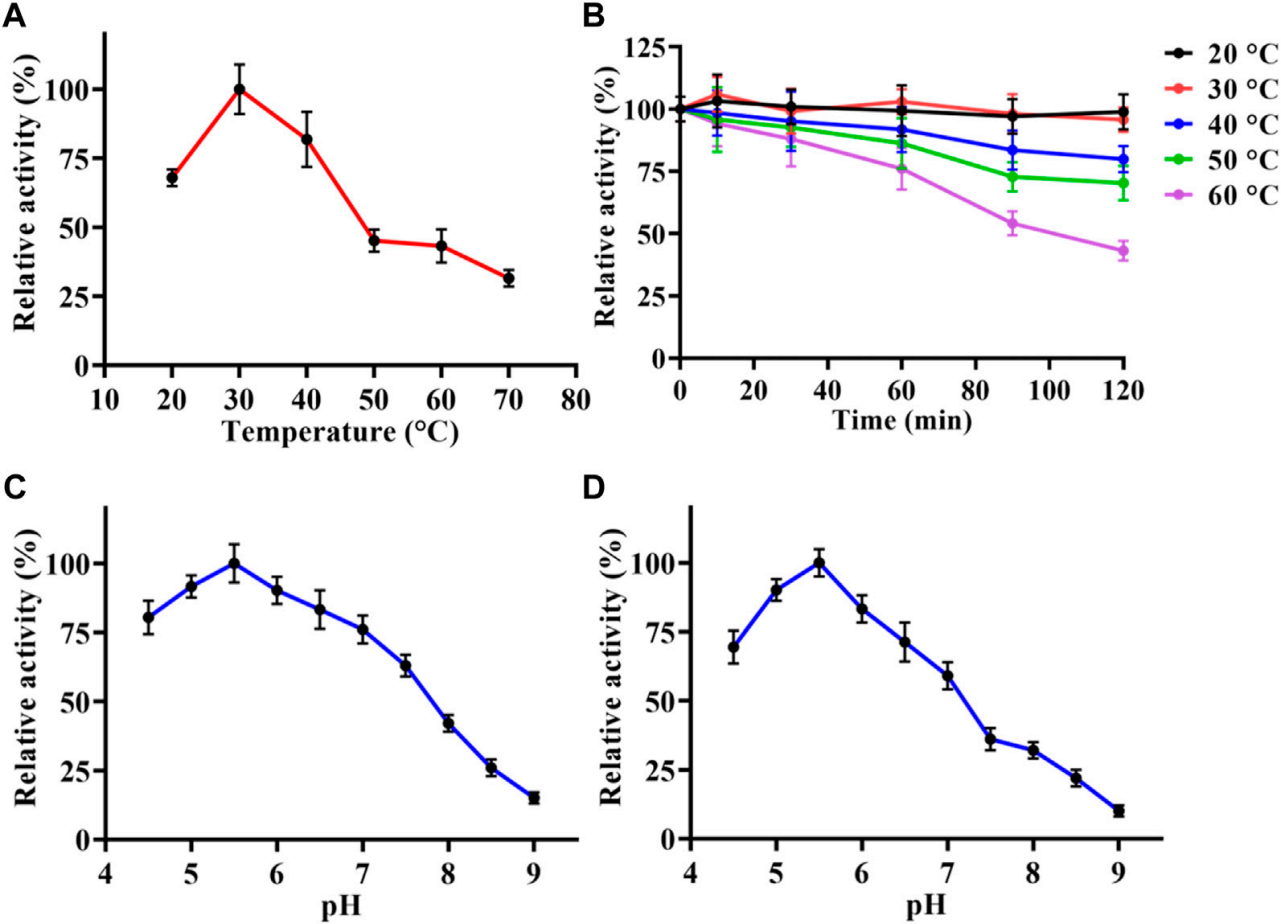
Enzymatic characterization of LpBgla. **(A)** Optimal temperature. **(B)** Thermostability analysis. **(C)** Optimal pH. **(D)** pH stability. The maximum activity of LpBgla is defined as 100% in each graph.

Temperature and pH were the key factors that affect the enzymatic activity. In order to analysis the enzymatic properties of LpBgla, we measured the β-glucosidase activities of purified LpBgla at different temperature and pH. As shown in Figure 2A, the maximum activity of LpBgla was defined as 100%, relative activities of this enzyme at 20°C–40°C were more than 60%. When the temperature was 30°C, the enzyme activity was maximized, suggesting that the optimal temperature of LpBgla was 30°C. After the LpBgla was incubated at 20°C–60°C for different times, the residual activities of all the samples were measured. In which, LpBgla still present approximate 100% of its initial activity after incubating at 20°C and 30°C for 120 min (Figure 2B), and retained over 70% activity when the temperature below 50°C (Figure 2B), indicating that LpBgla exhibited a good thermostability. LpBgla showed a general optimal temperature, which lower than some β-glucosidases from some other microorganisms, for example, *Aspergillus aculeatus* (70°C) (Li Y. et al., 2018), *Paecilomyces thermophila* (65°C) (Yan et al., 2012) and *Aspergillus fumigatus* Z5 (60°C) (Liu et al., 2012). The low optimal temperature maybe helpful for the application of LpBgla in the food industry.

LpBgla exhibited higher activity in acid conditions (pH < 7.0) than alkaline (pH > 7.0), in which, the optimal pH for enzyme activity was 5.5 (Figure 2C). The optimal pH of LpBgla was similar to some other β-glucosidases from *Aspergillus aculeatus* (pH 5.0) (Li Y. et al., 2018), *Clostridium cellulovorans* (pH 6.0) (Jeng et al., 2011) and *Aspergillus fumigatus* (pH 6.0) (Liu et al., 2012). We also found LpBgla could maintain high activities after treated in acid conditions (pH < 7) for 60 min, while the activities sharply decreased after incubated in alkaline solutions (pH > 7) (Figure 2D). Specially, the residual activity of LpBgla was maximum when treated at pH 5.5 for 60 min (Figure 2D). All these results indicated that LpBgla was an acid stable and resistant β-glucosidase, which could be used in fermentation food, cellulose hydrolysis processes, or some other acid environment industries.

**FIGURE 3:**
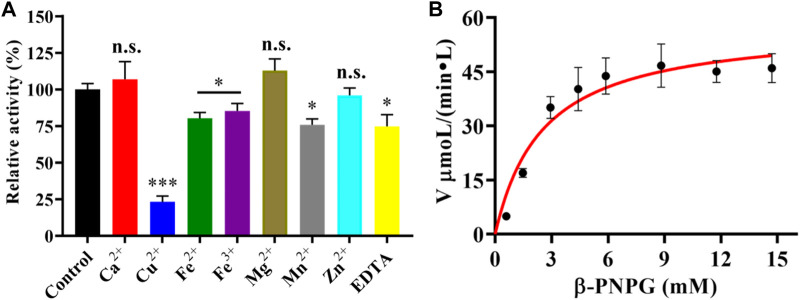
**(A)** Effect of various metal ions and EDTA on the enzymatic activity of LpBgla. The enzyme activity without any regents were defined as control (100%). All the values represent as Means ± SD (n = 3). n.s., not significant; *, *p* < 0.05; ***, *p* < 0.001. **(B)** Enzyme kinetics of LpBgla.

Moreover, we also investigated the effects of metal ions and EDTA on the purified LpBgla activity. As shown in Figure 3A, compared to the control, Ca^2+^ and Mg^2+^ increased the enzymatic activity to 107.3% and 112.9%, respectively. In the presence of Cu^2+^, Fe^2+^, Fe^3+^, Mn^2+^, and EDTA, the activity of LpBgla were significantly decreased (*p* < 0.05). Especially in the presence of Cu^2+^, the enzymatic activity decreased to 23.1% compared with the control (Figure 3A). Zn^2+^ showed negligible effects on the LpBgla activity (Figure 3A). It was indicated that several metal ions and agents, especially Cu^2+^, inhibited the enzymatic activity of LpBgla, suggesting that the active sites of this enzyme might have interactions with these metal ions, which made contributions to the enzymatic activity inhibition (Jeng et al., 2011; Liu et al., 2012).

### Kinetics analysis of LpBgla

The kinetic parameters of the purified LpBgla were determined using different concentrations of β-*p*NPG, and applying a nonlinear curve fit by GraphPad prism 8.0 software. The kinetics curve was shown in Figure 3B, based on the Michaelis-Menten calculations, the K_m_ value of LpBgla was 1.44 mM, and V_max_ value was 58.32 μM min^−1^. The k_cat_ was calculated, and showed a high enzyme turnover of 3.98×10^3^ s^−1^. Given by the k_cat_/K_m_ ratio of 2.77×10^3^ s^−1^ mM^−1^, the catalytic efficiency of LpBgla using β-*p*NPG as substrate was much higher than several other β-glucosidases, for example, from *Aspergillus fumigatus* (125.50 and 161.76 s^−1^ mM^−1^) (Liu et al., 2012), *Clostridium cellulovorans* (340 s^−1^ mM^−1^) (Jeng et al., 2011), *Micrococcus antarcticus* (1,120 s^−1^ mM^−1^) (Fan et al., 2011). Despite the monomeric nature of LpBgla, the sigmoidal character of the v_0_ vs. [S] remains and does not fit well to the hyperbola, it may be due to the conformational changes of enzyme’s activity center, or the substrate-induced coordination effects, or other enzyme-related factors to lead the atypical sigmoidal kinetic behavior (Porter and Miller, 2012). Similar to some previously reported monomeric enzymes with sigmoidal kinetics, for example, versatile peroxidase from *Bjerkendera adusta* (Siddiqui et al., 2014), cytochrome P450 enzyme 2C9 (Court et al., 2001) and glucokinase (Whittington et al., 2015) from human liver, et al., suggesting that sigmoidal kinetics are not only for oligomeric enzymes.

### Structural stability analysis of LpBgla at pH 7.5 or 5.5 by MD simulations

**FIGURE 4:**
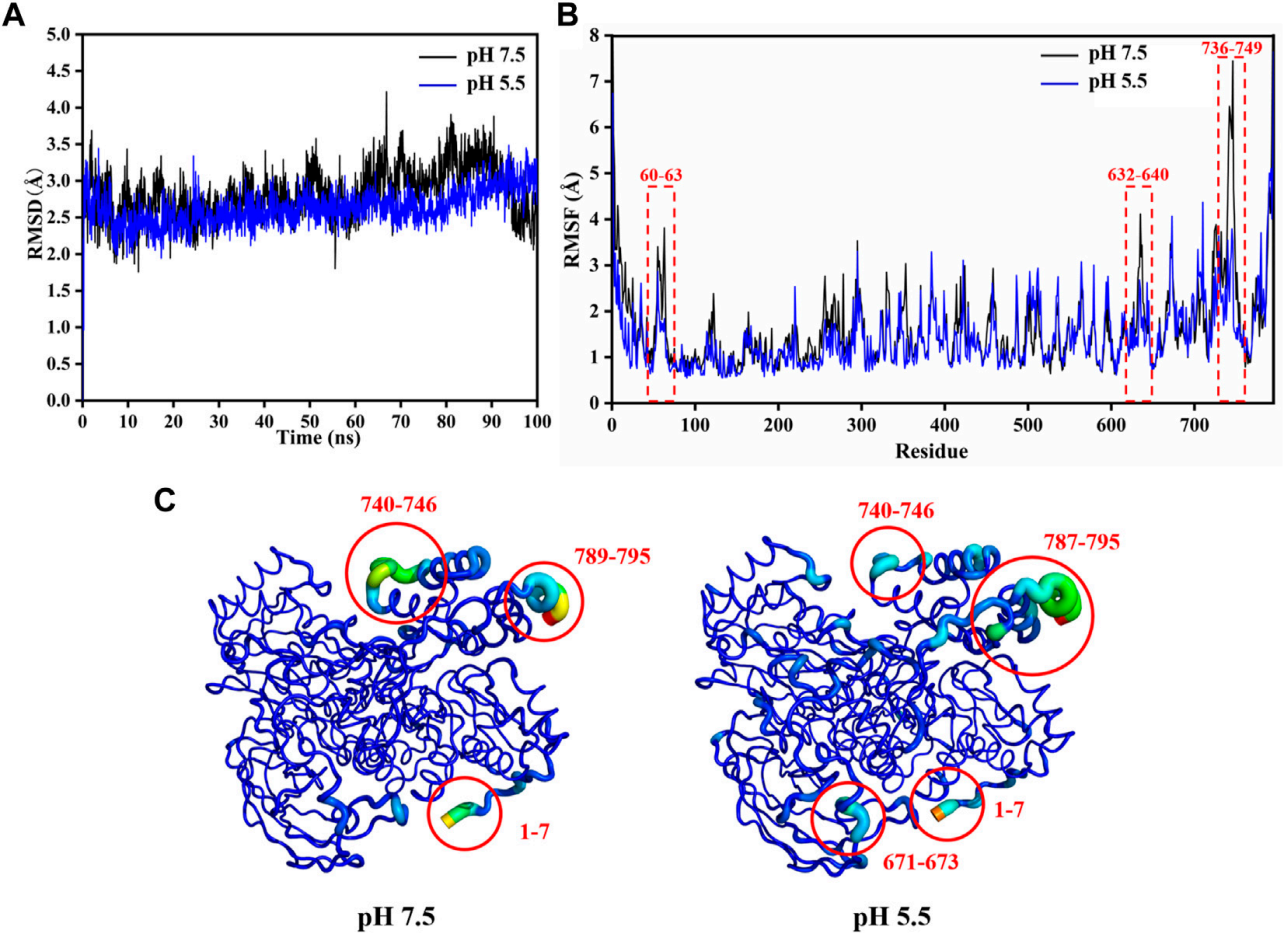
Structural stability analysis of LpBgla at different pH by MD simulations. **(A)** The root-mean-square deviation (RMSD) analysis. **(B)** The root-mean-square fluctuation (RMSF) value of each residue in the LpBgla at pH 7.5 and 5.5. **(C)** The B-factors of LpBgla at pH 7.5 and 5.5 binding to the substrate at the end of simulations.

Form the enzymatic property results, it was indicated that the LpBgla was an acid-adapted β-glucosidase, especially the optimal pH for its activity was 5.5. To investigate the involved molecular mechanism of the acid resistance of LpBgla, the structure and conformation changes of this enzyme at pH 5.5 and 7.5 were analyzed by MD simulations following our previously reported methods (Li Y. et al., 2018; Shang et al., 2022). As shown in Figure 4A, except the last 5 ns, the RMSD values of LpBgla at pH 5.5 were generally lower than that at pH 7.5 during the whole simulation time. Although the RMSF values of LpBgla at pH 5.5 and 7.5 were seemed similar, the RMSF at pH 7.5 were remarkably higher than that at pH 5.5 in residues of 60–63, 118–124, 632–640 and 736–749 (Figure 4B), indicating that the protein structure in these fragments were more flexible at pH 7.5 than at pH 5.5. Moreover, we also analyzed the average B-factor value of LpBgla residues at different pH, which would reflect the enzyme’s conformational state and structure flexibility. It was showed that the average B-factor value of LpBgla residues at pH 7.5 was higher than that at pH 5.5, suggesting that the LpBgla showed a more flexible conformation at pH 7.5 than at pH 5.5 (Figure 4C).

As the Rg value was on behalf of the density of protein system, we compared the Rg values of LpBgla at different pH. During the whole MD simulations, the Rg values of LpBgla at pH 7.5 were thoroughly higher than that at pH 5.5 (Supplementary Figure S4A). In line with the Rg results, the SASA values of LpBgla at pH 7.5 were also higher than that at pH 5.5 during the whole 100 ns simulation time (Supplementary Figure S4B). Therefore, all the above structural and conformational analysis indicated that structure of LpBgla at pH 5.5 was more stable than that at pH 7.5.

### Molecular interaction analysis between LpBgla and β-*p*NPG at pH 7.5 or 5.5 by MD simulations

**FIGURE 5:**
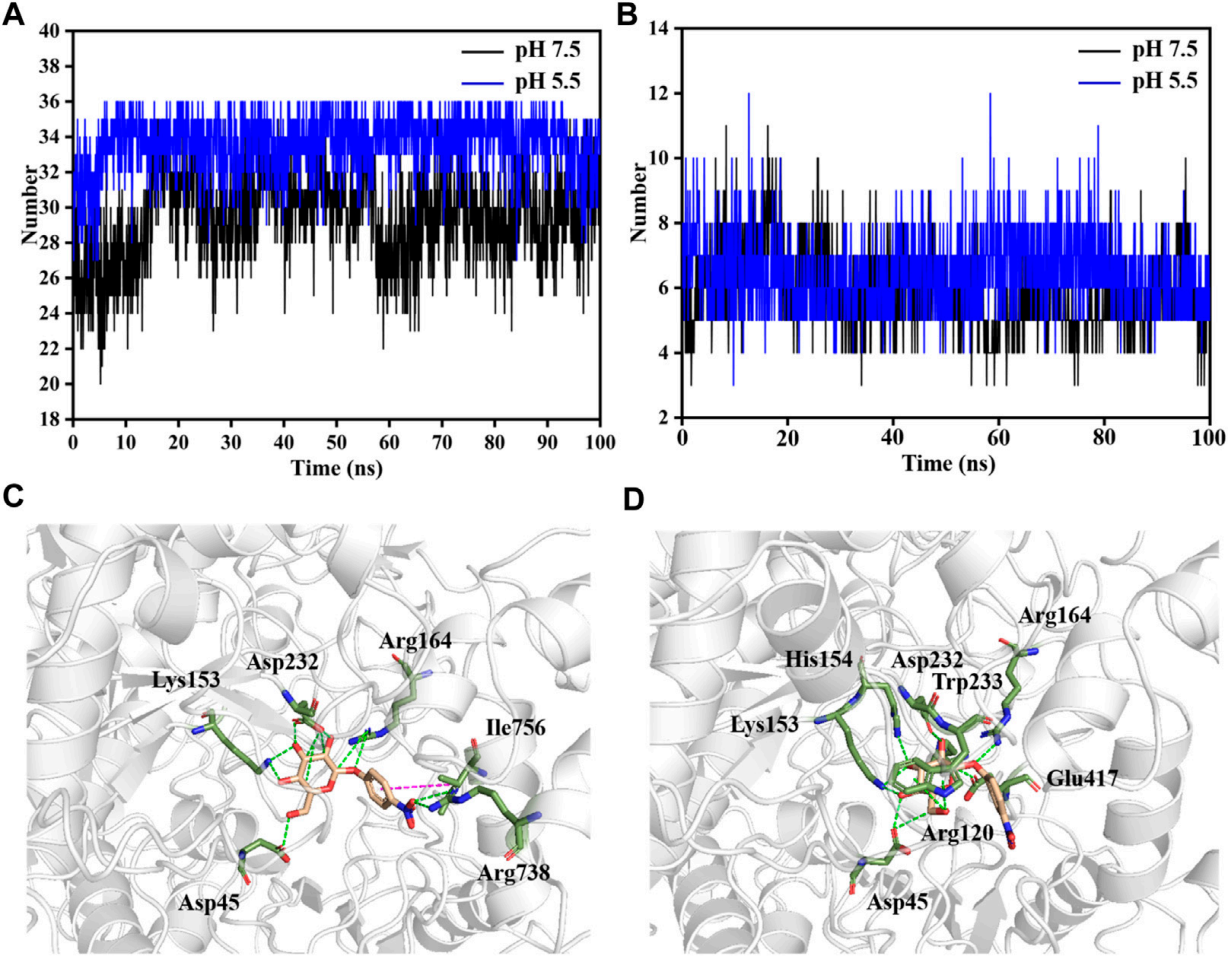
Analysis of molecular interaction between LpBgla and β-*p*NPG at different pH by MD simulations. **(A)** Average contact numbers, and **(B)** hydrogen bonds between LpBgla and substrate at pH 5.5 and 7.5 during the 100 ns simulations. Molecular interaction of LpBgla with substrate at **(C)** pH 7.5, and **(D)** pH 5.5 at the end of simulations. The hydrogen bonds were defined as green dashed lines, and Pi-Pi interaction was defined as magenta dashed line.

The interaction of LpBgla with the β-*p*NPG was important for the catalytic process. During the whole simulation time, although the minimum distance of LpBgla to the substrate was similar either at pH 7.5 or 5.5 (Supplementary Figure S5), the contact numbers between LpBgla and the substrate were different. As shown in Figure 5A, the average contact number during the simulation between LpBgla and the substrate at pH 5.5 was 33.6 ± 1.7, which was remarkable higher than that at pH 7.5 (29.5 ± 2.6). It was suggested that at low pH, more contacts were generated between the LpBgla and substrate, and in turn might cause more interactions. We furtherly analyzed the influence of different pH on the hydrophobic and electrostatic interactions, especially of the hydrogen bonds between LpBgla and substrate. Total number of hydrogen bonds of LpBgla at pH 7.5 and 5.5 were calculated. The data showed that there were 6.4 ± 1.1 hydrogen bonds between enzyme and substrate at pH 5.5 during the 100 ns simulation time, which was more than that at pH 7.5 (5.8 ± 1.2), indicating that the interaction of LpBgla with the substrate was stronger at pH 5.5 than pH 7.5 (Figure 5B).

The potential key residues that involved in the molecular interaction between LpBgla and β-*p*NPG were also investigated at different pH conditions. In the pH 7.5 system, six residues contributed main interactions of the enzyme with substrate (Figure 5C). In which, the β-*p*NPG interacted with the residues of Asp45, Lys153, Arg164, Asp232 and Arg738 by forming 10 hydrogen bonds, and interacted with Ile756 by forming Pi-Pi conjugate interaction (Figure 5C, S6A). Comparatively, eight residues including Asp45, Arg120, Lys153, His154, Arg164, Asp232, Trp233 and Glu417 involved in the interaction of LpBgla with the substrate at pH 5.5, and 12 hydrogen bonds were formed during the simulations (Figure 5D, S6B). There results demonstrated that more amino acids and hydrogen bonds were formed between LpBgla and β-*p*NPG at pH 5.5 than pH 7.5, suggesting that the interaction of the enzyme with the substrate at pH 5.5 is stronger than that at pH 7.5.

### Effects of different pH on the substrate-binding pocket of LpBgla by MD simulations

**FIGURE 6:**
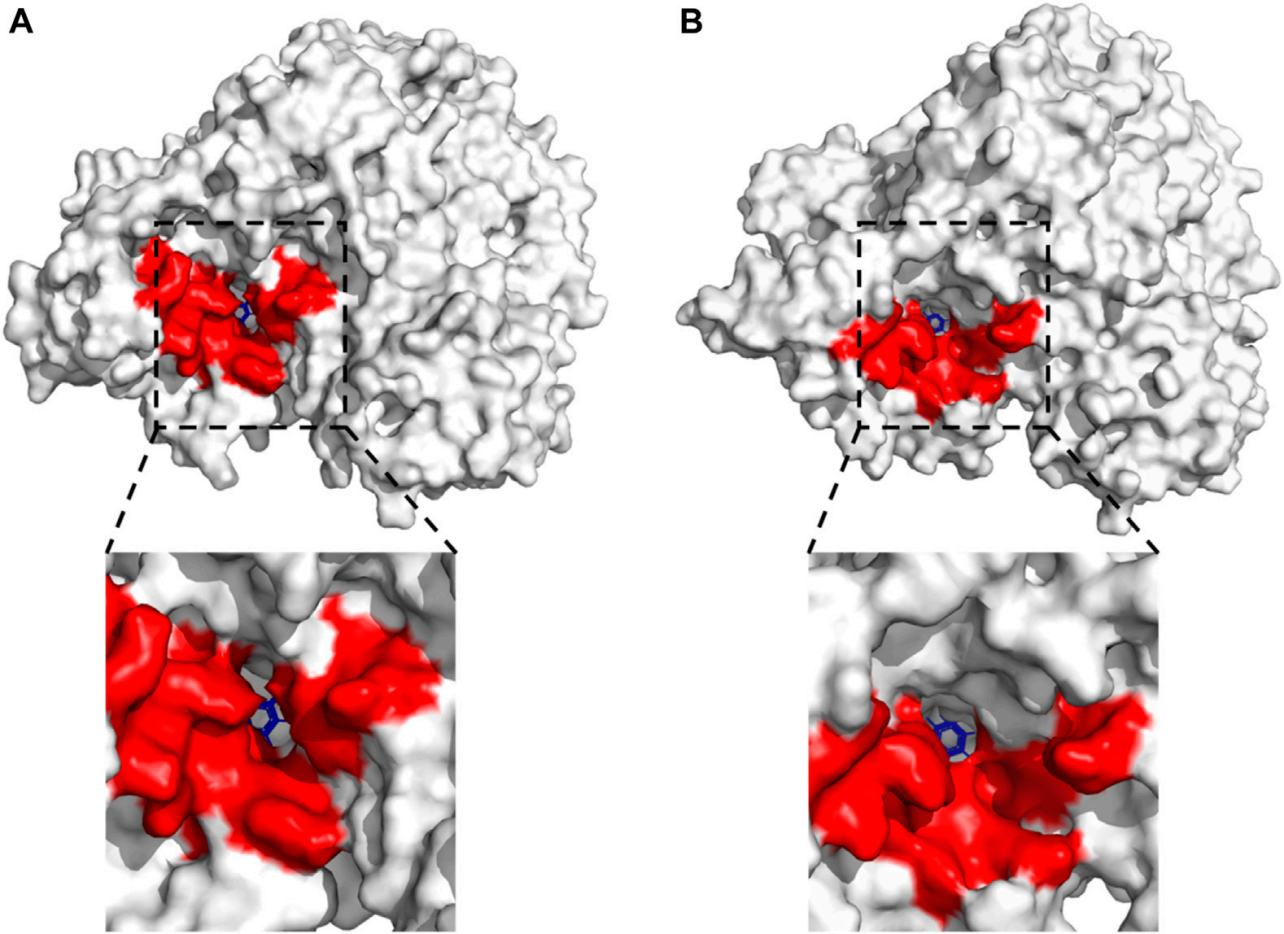
Effects of different pH on the substrate-binding pocket of LpBgla by DM simulations. Surface representation for the whole LpBgla structure at the last frame of simulations at **(A)** pH 7.5, and **(B)** pH 5.5. The below image in each panel is enlarged view of the substrate-binding pocket of LpBgla with β-*p*NPG.

It has been proved that the structure and interactions of LpBgla at pH 7.5 and 5.5 were obviously different, so in order to verify the effects of pH on the enzyme’s catalytic efficiency, the changes of internal structure around the substrate-binding pocket of the LpBgla-β-*p*NPG complexes were further analyzed. As shown in Figure 6A, at pH 7.5, there was only a relative narrow channel aisle for the accessing of substrate to the catalytic center. On the contrary, after the pH was 5.5, the substrate-binding pocket was enlarged in some degree, resulting a more open space and larger channel aisle for the substrate reaching the enzyme active center (Figure 6B). These structure changes on substrate-binding pocket will not only expose the available space inside the active center, but also decrease the steric hindrance during the substrate accessing. The results fit the concept that to improve the enzymes’ activity by modifying the surrounding amino acids to enlarge their substrate-binding pocket in lots of literature (Li et al., 2022; Jia et al., 2023; Li et al., 2023). It was suggested that the substrate-binding pocket and channel aisle of LpBgla might have enlarged at pH 5.5 compared to pH 7.5.

### Binding free energy analysis and residue contributions of LpBgla to β-*p*NPG at different pH by MD simulations

Based on the MD simulations, the binding free energy of LpBgla to the β-*p*NPG at pH 7.5 and pH 5.5 were calculated, and the binding free energy and contributions of its ingredients for enzyme-substrate complex were listed in Table 1. As the calculation results, electrostatic interaction and van der Waals energy in the complex were favorable for the binding (Table 1). The binding free energy between the LpBgla and β-*p*NPG at pH 7.5 was −62.83 ± 18.53 kJ/mol, while the binding free energy was −106.91 ± 18.17 at pH 5.5, indicating that LpBgla had a stronger binding affinity to β-*p*NPG at pH 5.5 than at pH 5.5 (Table 1).

**TABLE 1:** Binding free energy between LpBgla and β-*p*NPG at pH 7.5 and pH 5.5.

Energetic component	7.5	5.5
∆*G*_bind_ (kJ/mol)^a^	−62.83 ± 18.53	−106.91 ± 18.17
∆*E*_vdw_ (kJ/mol)	−87.53 ± 20.19	−111.92 ± 19.62
∆*E*_ele_ (kJ/mol)	−229.52 ± 33.29	−264.55 ± 17.62
∆*G*_solv_ (kJ/mol)	269.82 ± 41.19	286.34 ± 17.80
∆*G*_SASA_ (kJ/mol)	−15.60 ± 2.23	−16.78 ± 0.80

^a^ The binding free energy (∆G_bind_) was calculated by the summation the values of the van der Waals energy (∆*E*_vdw_), electrostatic energy (∆*E*_ele_), polar solvation energy (∆*G*_solv_), and solvent accessible surface areas energy (∆*G*_SASA_).

**FIGURE 7:**
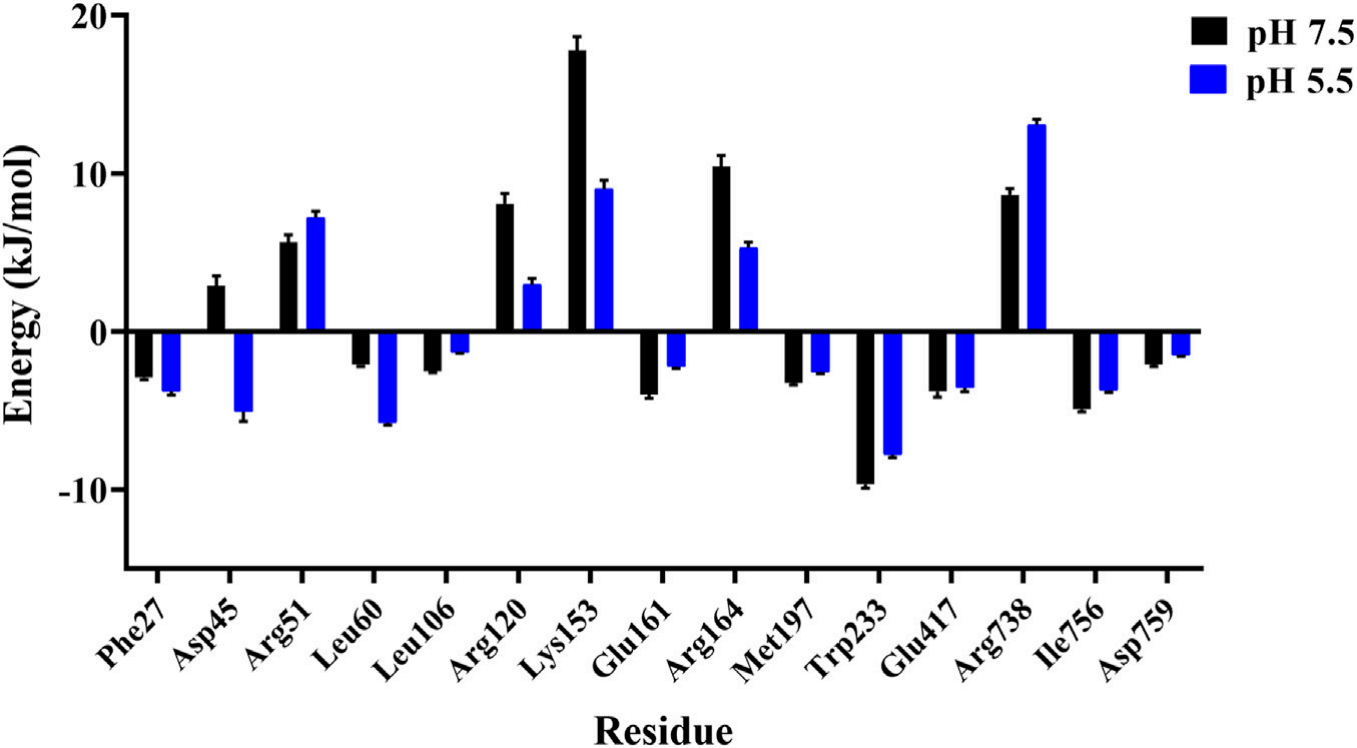
Binding free energy contribution of each residue of the LpBgla in the complex with β-*p*NPG at pH 7.5 and pH 5.5.

Moreover, we also probed the contributions of each residue for binding free energy in LpBgla to β-*p*NPG at different pH. 15 residues with the highest absolute binding free energy in LpBgla were screened (Figure 7). In which, at pH 5.5, residues of Asp45, Leu60, Arg120, Lys153 and Arg164 showed significantly lower binding energy than that pH 7.5 (Figure 7), contributing the improvement on binding affinity of LpBgla to the substrate. Therefore, the binding free energy and decomposition energy calculation data were consistent to the structural and interaction results, indicating that LpBgla showed a stronger binding affinity to β-*p*NPG at pH 5.5 than pH 7.5, and the five residues (Asp45, Leu60, Arg120, Lys153 and Arg164) might play a critical role in the acid-adapted mechanism of LpBgla.

### Potential application of LpBgla in glycosides bioconversion and specificity analysis

**FIGURE 8:**
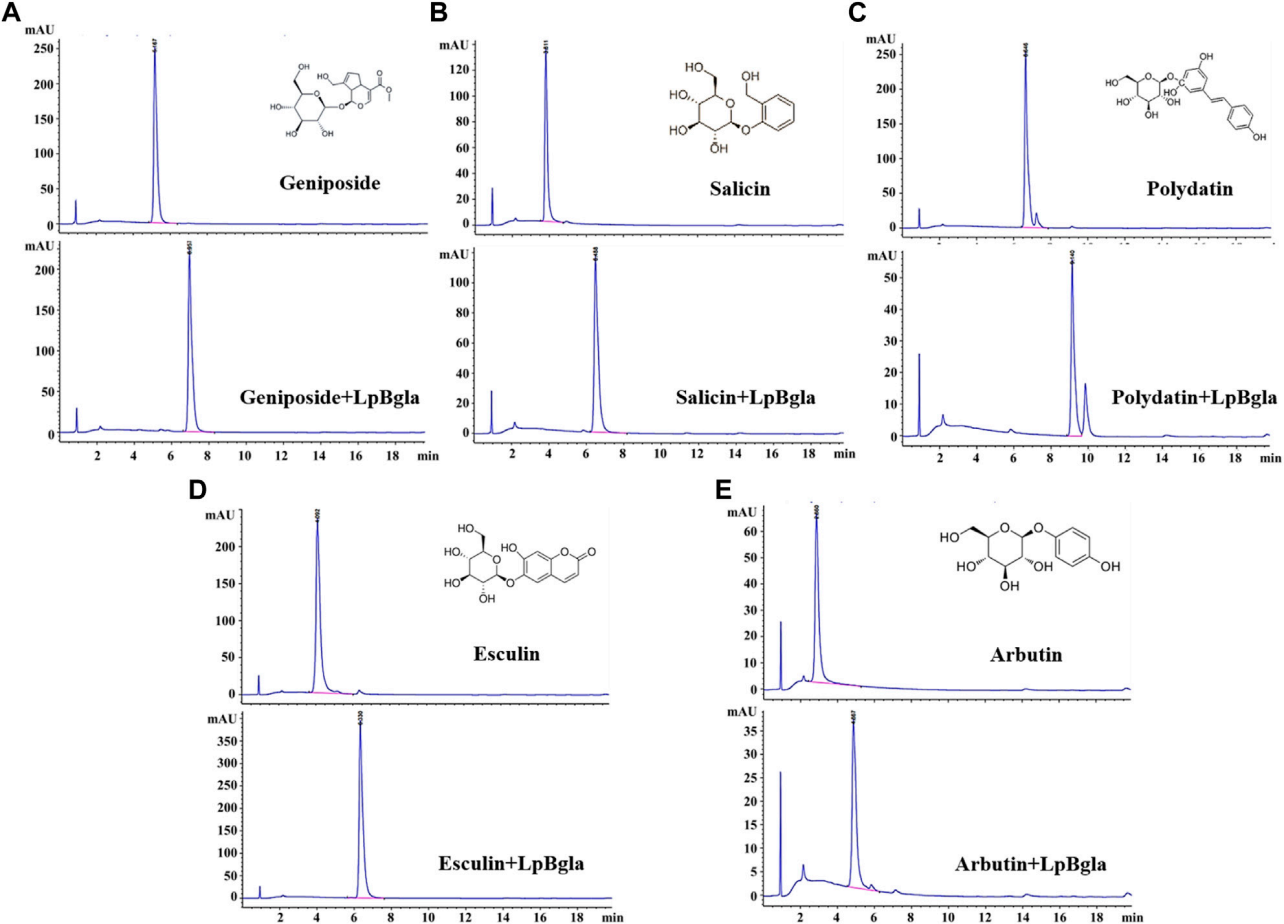
Bioconversion of various glycosides using LpBgla. **(A)** Geniposide. **(B)** Salicin. **(C)** Polydatin. **(D)** Esculin. **(E)** Arbutin.

It has been reported that β-glucosidase can be used in the bioconversion of glycosides to produce aglycones (Bhatia et al., 2002; Yuksekdag et al., 2018; Xie et al., 2022). And the aglycones possess higher bioactivity than glycosides because of the reducing glycosyl groups, lower molecular weight and more easily absorbed by the gut (Harada et al., 2005; Xiao, 2017). Therefore, we explored the potential bioconversion effects of LpBgla on various glycosides, including geniposide, salicin, polydatin, esculin and arbutin. After catalysis by LpBgla, all the five glycosides conversed to lower molecular weight compounds from the HPLC results (Figure 8), indicating that LpBgla could be applied in the bioconversion of glycosides, and with a broad substrate specificity. The β-glucosidases have been grouped into three classes based on the catalytic substrate: (1) aryl-β-glucosidases, (2) true cellobiases, and (3) active on broad substrate specificity enzymes. It is was reported that most of the characterized β-glucosidases acted on a wide spectrum of substrates and belonged to the third category broad substrate specificity enzymes (Bhatia et al., 2002). For example, both of the β-glucosidases from *Sulfolobus solfataricus* and *Pyrococcus furiosus* showed a broad specific activity towards isoflavone glycosides, including daidzin, glycitin, genistin, malonyl genistin, malonyl daidzin, malonyl glycitin (Kim et al., 2012; Yeom et al., 2012). Except towards the aryl-glycosides, extracellular β-glucosidase from *Paecilomyces thermophila* J18 exhibited a broad substrate specificity and significantly hydrolyzed towards oligosaccharides and some β-glucans (Yang et al., 2008). Li et al., also reported a thermostable GH3 β-Glucosidase, Bgl3B from *Talaromyce leycettanus* with substrate specificity towards genistin and daidzin, but also with a broad substrate specificity towards oligosaccharides and polysaccharides (Li X. et al., 2018). Evern for the β-glucosidase from human, after heterogeneously expressed in *Pichia pastoris*, the purified enzyme showed broad substrate specificity with respect to the aglycone moiety of various aryl-glucosides, similar to the native enzyme (Berrin et al., 2002). Currently, lots of studies are underway to probe the molecular basis and mechanism of β-glucosidases’ wide substrate specificity. Identification of amino acids that occurring at the active site and substrate binding pocket are of considerable importance for revealing the enzyme’s structure-function relationships, which is also beneficial to improve the enzyme characteristics by designing and constructing mutants (Bhatia et al., 2002).

To further analysis the substrate specificity of LpBgla on different glycosides, the enzyme kinetics of LpBgla on geniposide, salicin, polydatin, esculin and arbutin were analyzed. In a general enzymatic reaction case, the index of k_cat_ often referred to as the catalytic rate of an enzyme, a higher k_cat_ value implies a higher catalytic rate. K_m_ is used to reflect the affinity between the enzyme and substrate, in which, a lower K_m_ value indicates a higher affinity of enzyme to the substrate. The k_cat_/K_m_ can be used to reflect one enzyme’s catalytic efficiency, or used as a specificity constant to compare the catalytic efficiency of one enzyme to different substrates (Eisenthal et al., 2007). The corresponding kinetic parameters were listed in Table 2, k_cat_ values of LpBgla on geniposide, salicin, polydatin, esculin and arbutin were 2,520, 2,973, 1728, 2,207 and 3,569 s^−1^, respectively, indicating that the LpBgla showed the highest enzymatic rate on arbutin among these five substrates, while lower than β-*p*NPG. The K_m_ values of LpBgla on geniposide, salicin, polydatin, esculin and arbutin were 2.87, 2.25, 5.71, 3.61 and 1.92 mM, respectively, suggesting the affinity between LpBgla and arbutin was the strongest among these five substrates. The k_cat_/K_m_ values of LpBgla on the above five substrates were 878, 1,321, 303, 611 and 1859 s^−1^ mM^−1^, respectively (Table 2). In line with the substrate affinity results, the catalytic efficiencies of LpBgla towards arbutin was the highest among these five substrates, which was about 67.2% compared to the β-*p*NPG (Table 2).

**TABLE 2:** Kinetic parameters of purified LpBgla.

Substrate	K_m_ (mM)	k_cat_ (s^−1^)	k_cat_/K_m_ (s^−1^ mM^−1^)
β-*p*NPG	1.44 ± 0.08	3,982 ± 14	2,765
Geniposide	2.87 ± 0.05	2,520 ± 23	878
Salicin	2.25 ± 0.01	2,973 ± 88	1,321
Polydatin	5.71 ± 0.10	1728 ± 45	303
Esculin	3.61 ± 0.08	2,207 ± 38	611
Arbutin	1.92 ± 0.03	3,569 ± 72	1859

## Conclusion

In this study, the LpBgla was cloned and characterized from *Lactobacillus paracasei* TK1501. The recombinant LpBgla was an acid-adapted enzyme that exhibited maximal activity at temperature of 30°C and pH 5.5, and the enzymatic activity was inhibited by Cu^2+^, Mn^2+^, Zn^2+^, Fe^2+^, Fe^3+^ and EDTA. The K_m_ value of LpBgla was 1.44 mM, and the catalytic efficiency given by the k_cat_/K_m_ ratio was 2.76×10^3^ s^−1^ mM^−1^. MD simulations indicated that LpBgla showed more stable structure, and wider substrate-binding pocket and channel aisle at pH 5.5 than pH 7.5. The molecular interaction between LpBgla and β-*p*NPG at pH 5.5 was stronger than pH 7.5 due to the additional hydrogen bonds and lower binding free energy. Five residues including Asp45, Leu60, Arg120, Lys153 and Arg164 might play a critical role in the acid-adapted mechanism of LpBgla. Moreover, LpBgla showed a broad substrate specificity and potential application in the bioconversion of glycosides, especially towards the arbutin.

## Data Availability

The datasets presented in this study can be found in online repositories. The names of the repository/repositories and accession number(s) can be found in the article/Supplementary Material.
