# Supplementary material for: Characterization and insight mechanism of an acid-adapted β-Glucosidase from Lactobacillus paracasei and its application in bioconversion of glycosides
Source: Front Bioeng Biotechnol. 2024 Jan 24;12:1334695. doi: 10.3389/fbioe.2024.1334695 (PMC10851751; doi:10.3389/fbioe.2024.1334695)
Supplement: Supplementary file 1 [file DataSheet1.docx]

**Cover Sheet for Supporting Information**

**Characterization and insight mechanism of an acid-adapted β-****Glucosidase from *Lactobacillus paracasei* and its application in bioconversion of glycosides**

**Yufeng Xie^1,2^, Xinrui Yan^1^, Changzhuo Li^1^, Shumei Wang^1^*****, Longgang Jia^2^***

^1^ College of Food Science and Engineering, Harbin University, Harbin 150086, China

^2^ Tianjin University of Science and Technology, Tianjin 300457, China

* Corresponding author:

E-mail address: [longgangjia@tust.edu.cn](mailto:longgangjia@tust.edu.cn) (L. Jia); [wangshumei24@126.com](mailto:wangshumei24@126.com) (S. Wang).

Tel.: +86 022 60602949; Fax: +86 022 60602298.

Postal and addresses for all authors:

1 No. 109 Zhongxing Avenue, Harbin City, Heilongjiang Province, 150086, China

^2^ No.29, 13th Avenue, Tianjin Economic and Technological Development Area, Tianjin 300457, China

Number of pages: 5 (including the cover sheet)

Number of supplemental tables: 0

Number of supplemental figures: 4

**Supplemental methods**

**Enzymatic Kinetic Analysis**

The kinetic analysis of LpBgla on β-*p*NPG was performed by measuring the enzyme activity at 30 °C, pH 5.5 for 30 min. The concentrations of substrate were 0.59, 1.47, 2.94, 4.41, 5.88, 8.82, 11.76 and 14.71 mM. The *K_m_* and *V_max_* values were determined by fitting the data to the Michaelis-Menten equation, and the *k_cat_* was calculated by *k_cat_*=*V_max_*/(Cenc.). All the data were processed using GraphPad prism 8.0 software. The Michaelis-Menten equation was obtained as: *v_0_*=*V_max_*[S]/(*K_m_*+[S]), in which, *v_0_* was the initial rate of enzymatic reaction; *V_max_* was the maximum rate of enzymatic reaction, 57.99 mM/min; [S] was the concentration of substrate; *K_m_* was the dissociation constant, 2.53 mM.

**Molecular Mechanics/Poisson Boltzmann surface Area (MM/PBSA) calculation**

The binding free energy of LpBgla with the β-*p*NPG was calculated using the MM/PBSA with g_mmpbsa tool following previously reported method ([Miller et al., 2012](#_ENREF_2); [Kumari et al., 2014](#_ENREF_1)). Based on the MD trajectory, the binding energy within 0-10 ns was calculated by g_mmpbsa tool and Numpy software (Numerical Python). The binding free energy is calculated following the Equation (1):

∆G_bind_ = ∆E_vdw_ + ∆E_ele_ + ∆G_solv_ + ∆G_SASA_ (1)

Among them, ∆G_bind_ is the binding free-energy, ∆E_ele_ is the electrostatic interaction, ∆E_vdw_ is the van der Waals force, ∆G_SASA_ is the non-polar contribution energy, and ∆G_solv_ is the solvent free-energy.

**Supplementary references**

Kumari, R., Kumar, R., Lynn, A. (2014). g_mmpbsa--a GROMACS tool for high-throughput MM-PBSA calculations. J. Chem. Inf. Model., 54: 1951-1962. 10.1021/ci500020m

Miller, B. R., 3rd, Mcgee, T. D., Jr., Swails, J. M., Homeyer, N., Gohlke, H., Roitberg, A. E. (2012). MMPBSA.py: An Efficient Program for End-State Free Energy Calculations. J. Chem. Theory Comput., 8: 3314-3321. 10.1021/ct300418h

**Table S1**. Amino acid sequence of LpBgla from *Lactobacillus paracasei* TK1501.

| **Protein name** | **Amino acid sequence** |
| --- | --- |
| LpBgla | MGVVVSNFHLAKITAEEKVKLTSGKDFWTSEHLADKGIPSFRMSDGPHGLRYQALAADHLGINDSVPSTSFPTASASAAAWDPDLIQAMGKAIGLEAQSLGVDMVLGPGVNMKRNPLCGRNFEYFSEDPFLAGKLGAAWINGIQSQGIAACLKHFAANNQENDRLSSDSLVDPTALHEIYLEAFRIAVTESHPEAVMCSYNKINGTYASDNLYLMTQVLRQQFGFGGAVITDWGALNDKVAALNAGTDLEMPGDDHLFDGEALQAYQQGTLKLASLDRAVTKIAEIARKQRPKFQGSREQLLQANGQLAQKIAESAIVLLKNEAALLPLQATDTVAVIGELAKATRFQGAGSSHINASEIVSVLDGLKQKKVSFDYAAGYRLDDQDDSQATAEALALARNHDKVVFVAGLPDNYESEGFDRQNMALPKVQNDLLQAVTAVNPNVIVLLVAGAPVELPWVDQVKAVVNLSLGGERIGAAAANVLTGAVNPSGKLAESYPLKYQDVPSADVYDKKPRSVPYVESTYIGYRYYDKAKVPVAFPFGFGLSYTSFALKNIQLSSDHVTDDQPLTISLQVTNTGQVDGAEVVQVYVQEQQPRPLRPEKSLKAFKKVFVKAGQTVNVALELKAQAFKEWREQTQTWVLPEAQKAIAVGTSVTNIDAVLPVSFTGETFNNFATIPNWYTTLSGKPSVQDFEQLTDQKVPAPHEFVPGEFTRLNTPREMKKHSLLLRLVAWITVKIRTKDYIDKQGPEAKFQQAIVLDTPLIRLAQQASGALKLSMVDRLVAAANHQYVKMIFR |


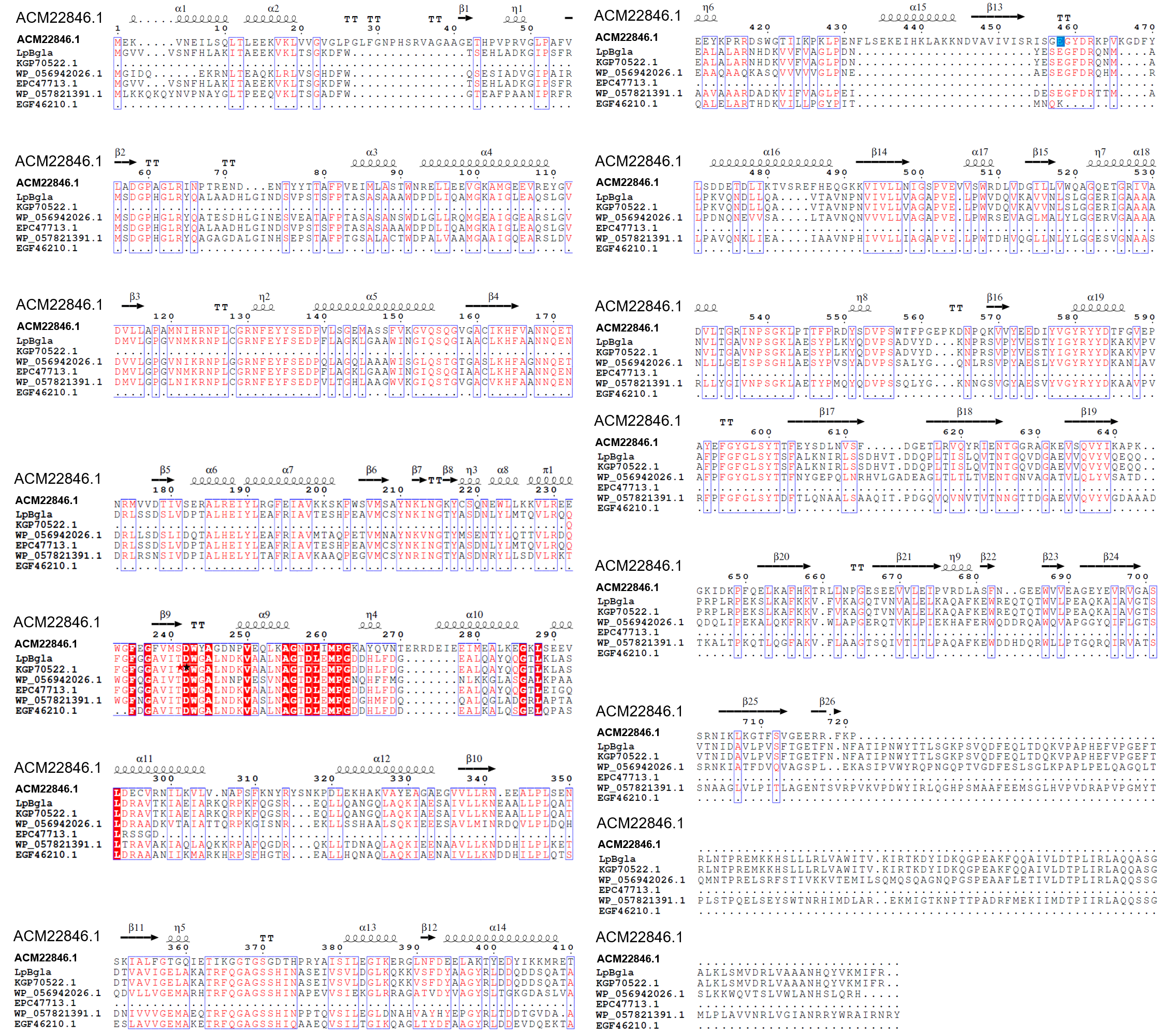


**Figure S1.** The complete multiple sequence alignment of LpBgla with Bglas from *Lactobacillus casei* (KGP70522.1), *Lactobacillus perolens* (WP_057821391.1), *Lactobacillus kimchicus* (WP_056942026.1), *Lactobacillus paracasei* (EPC47713.1), *Thermotoga neapolitana* (ACM22846.1) and *Lactobacillus rhamnosus* (EGF46210.1).


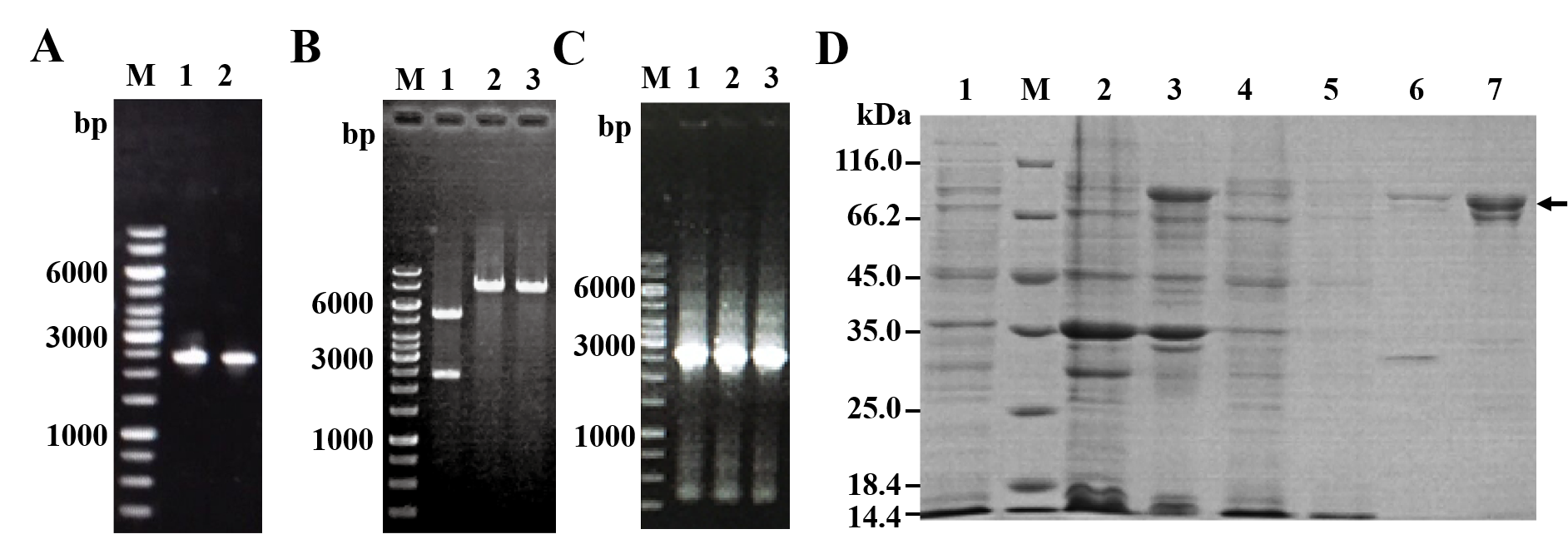


**Figure S2.** Cloning of the LpBgla in *E. coli*. (A) Cloning of the *LpBgla* gene using the *Lactobacillus paracasei* TK1501 genome DNA as template. M, DNA marker; lines 1-2 are two PCR samples. (B) Identification of the pET28a-*LpBgla* by restriction enzyme digestion. M, DNA marker; line 1, the plasmid double digested by *Hin*dIII and *Xho*I enzymes; lines 2 and 3, the plasmid individually digested by *Hin*dIII and *Xho*I, respectively. (C) Identification of the BL21-*LpBgla* using colony PCR. M, DNA marker; lines 1-3 are three transformants. (D) Purification of LpBgla using Ni-NTA affinity chromatography. M, protein molecular weight marker; line 1, supernatant of BL21-LpBgla after induced by IPTG; lines 2 and 3, pellet and supernatant of BL21-LpBgla after lysis by sonication; line 4, flow-through of the soluble protein from column; line 5, wash buffer; lane 6, residual protein in the resin; line 7, elution buffer.





**Figure S3.** Purification and identification of the LpBgla monomer using size-exclusion chromatography. The black lines are standard protein markers of 75 and 158 kDa, the red line is the LpBgla.


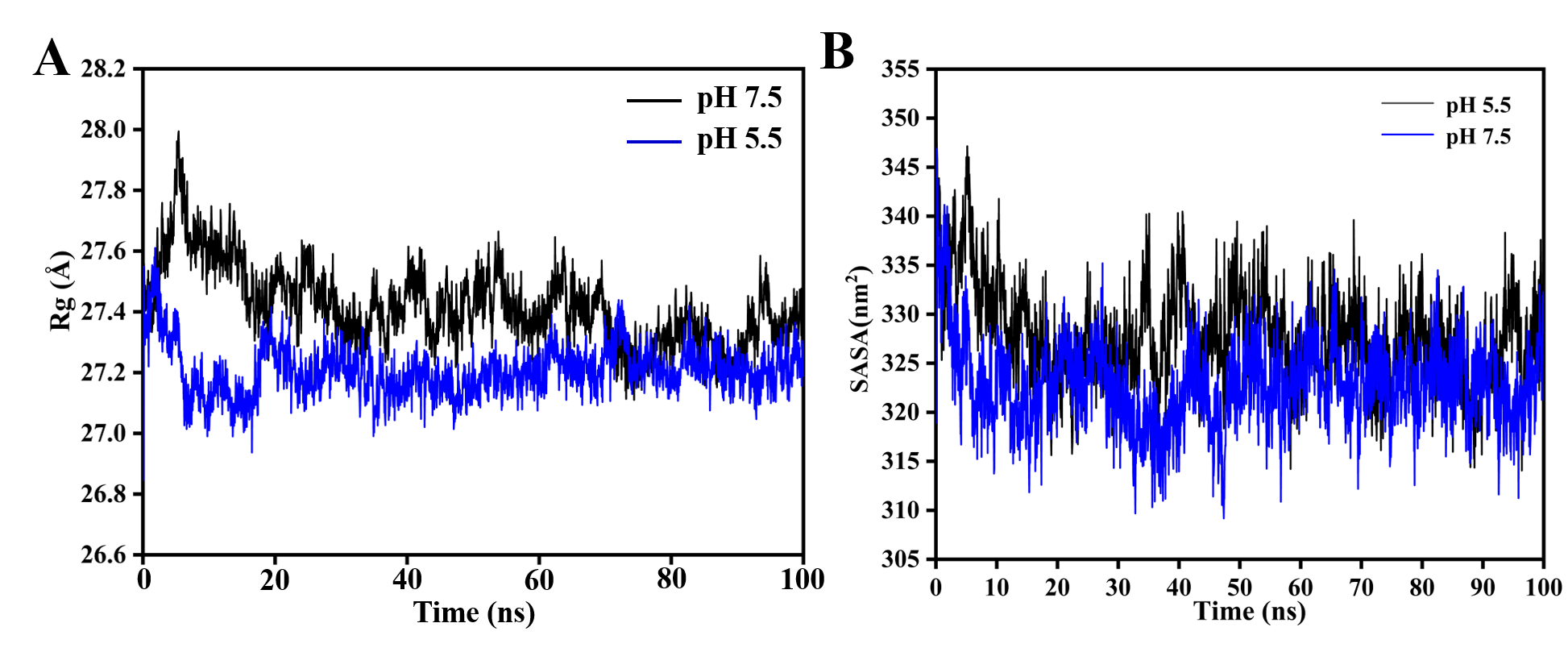


**Figure S4.** (A) The radius of gyration (Rg) and (B) the solvent accessible surface area (SASA) values of LpBgla at pH 7.5 and 5.5 binding to the β-*p*NPG during the simulations.


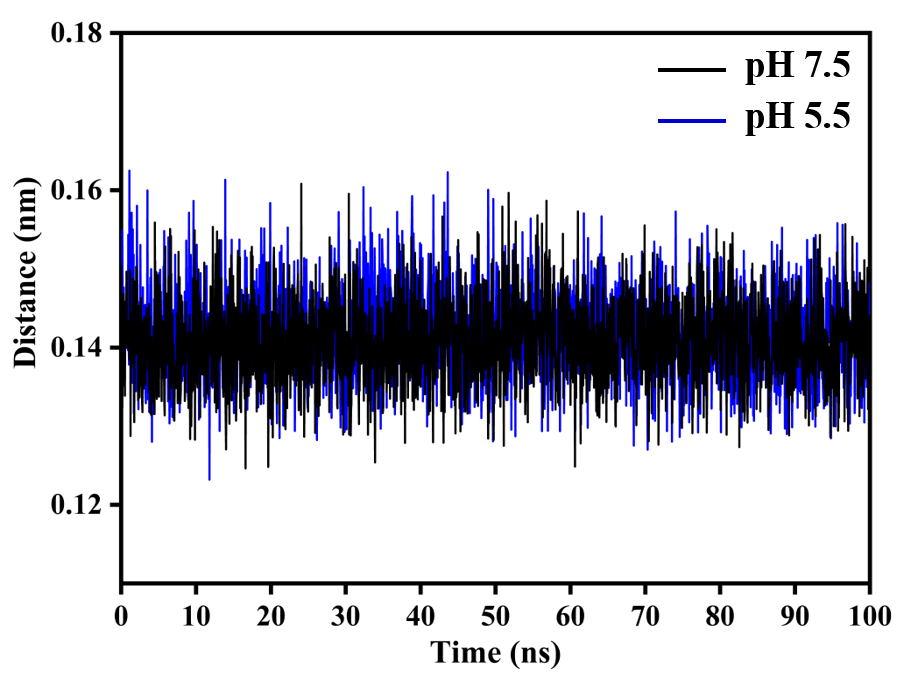


**Figure S5.** Distance between LpBgla and β-*p*NPG at pH 7.5 and pH 5.5 during the 100 ns simulations.


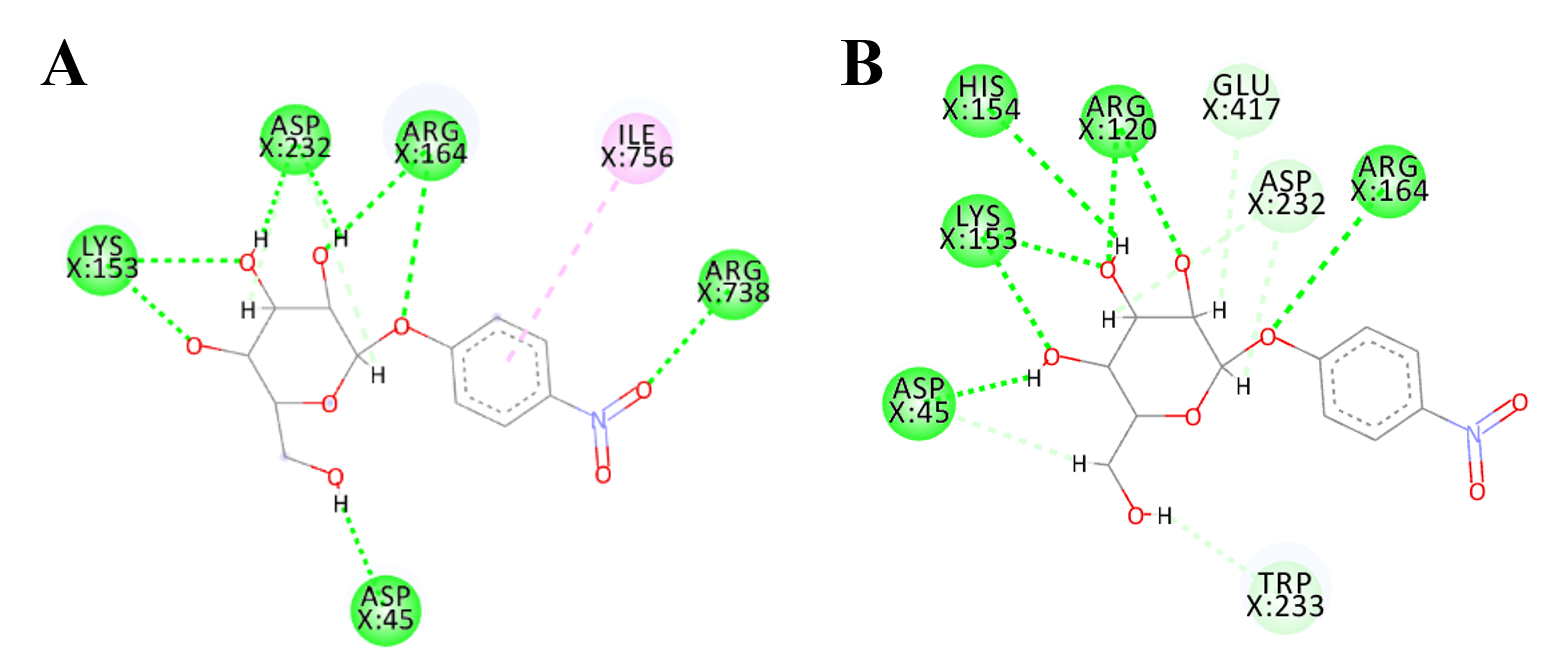


**Figure S6.** Molecular interaction between LpBgla and β-*p*NPG at (A) pH 7.5, and (B) pH 5.5 at the end of simulations. The hydrogen bonds were defined as green dashed lines, and Pi-Pi interaction was defined as magenta dashed line.
